# Supplementary material for: Balance training in older adults enhances feedback control after perturbations
Source: PeerJ. 2024 Nov 25;12:e18588. doi: 10.7717/peerj.18588 (PMC11604044; doi:10.7717/peerj.18588)
Supplement: Supplemental Information 1 [file peerj-12-18588-s001.docx]

# **Supplementary material**

## **Training program**

| Table S1: Training program progression plan. Exercises further down the list are increasingly difficult. Progression to more difficult exercises was based on the physical therapist’s observation during the training sessions; if the participant was able to perform the task for 60 seconds, the difficulty would be increased. | | |
| --- | --- | --- |
| **Exercises** | | **Duration/Frequency** |
| Warm-up: Head rotations, back stretching, trunk rotations | |  |
| Balancing   - one leg stance - switch legs - unstable surfaces | | 3 x 60 seconds  2 repetitions |
| Balancing, eyes-closed   - one leg stance (when possible) - switch legs - unstable surfaces | | 3 x 60 seconds  2 repetitions |
| Displacement of weight   - one leg stance - switch legs - unstable surfaces | | 3 x 60 seconds  2 repetitions |
| Passing/throwing around a ball in groups of 4 | | 5 rounds, both directions  3 repetitions |
| - fitness ball   - one leg   - unstable surface | - 2 kg ball   - one leg   - unstable surface |  |
| Alternative approaches for more trunk rotation:   - bigger circle - backs towards each other | |  |
| Pass big ball around: stop ball with foot and roll it back | | 5 rounds, both directions  3 repetitions |
| - fitness ball   - one leg   - unstable surface | - 2 kg ball   - one leg   - unstable surface |  |

| *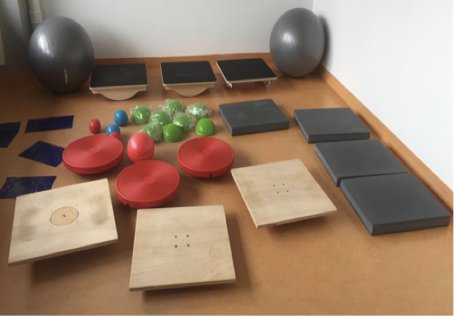* |
| --- |
| Figure S1: Equipment used during the training sessions. |

## **Experimental setup and model representation**

| 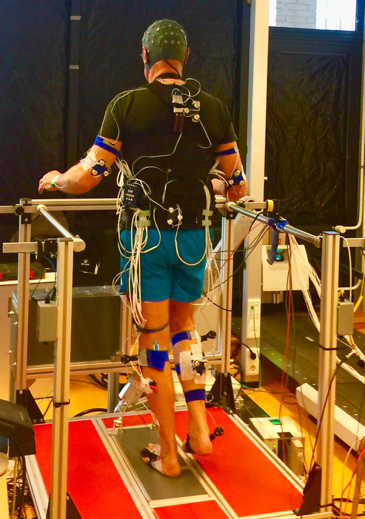 | 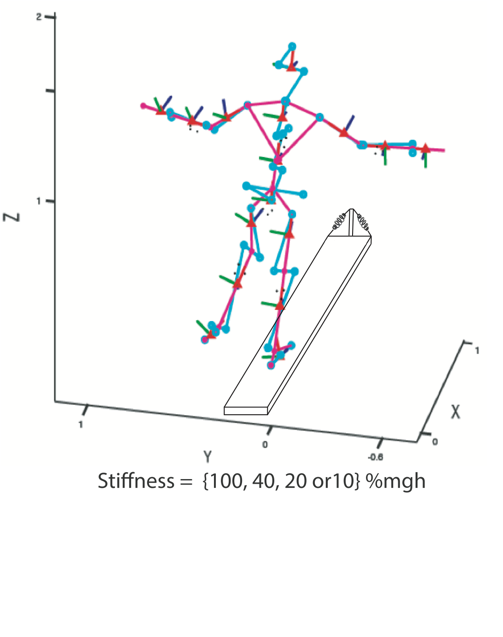 |
| --- | --- |
| Figure S2.1: The experimental setup of a participant in unipedal stance on the robot-controlled mediolateral rotating platform. | Figure S2.2: Example of the model, constructed from the cluster markers, from which the kinematic parameters are obtained. |

## **Complete overview statistics – Lateral perturbation**

| **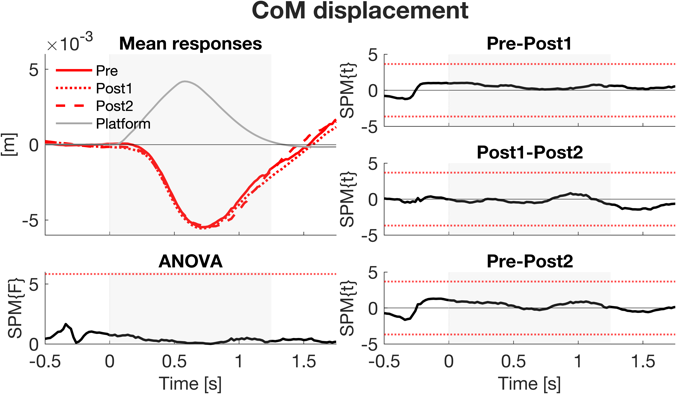 A** | **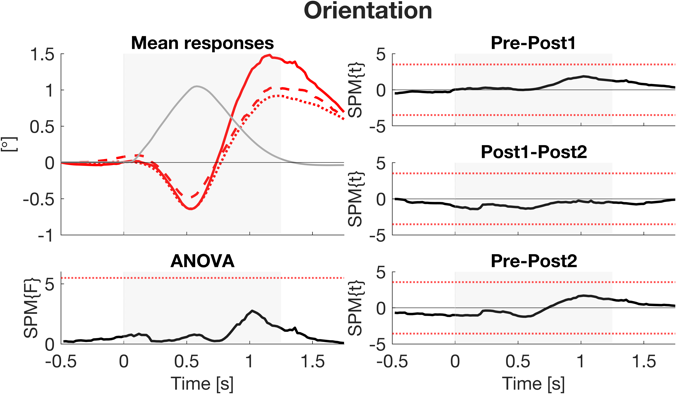 B** |
| --- | --- |
| **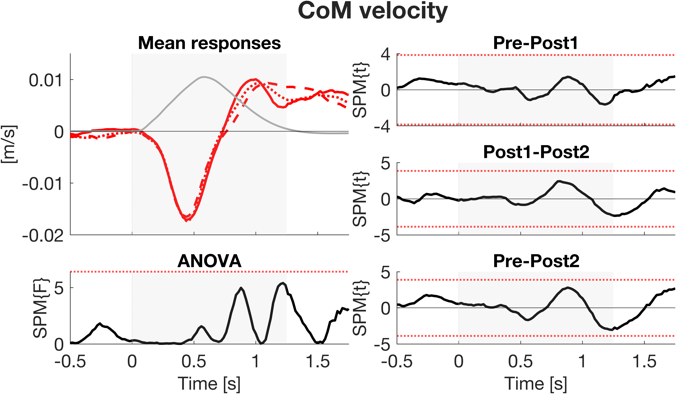 C** | **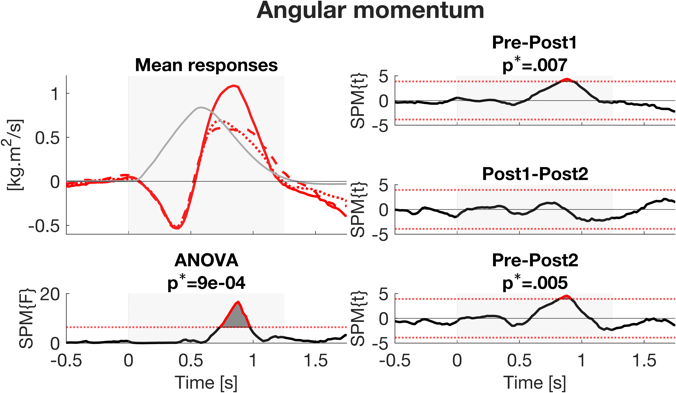 D** |
| **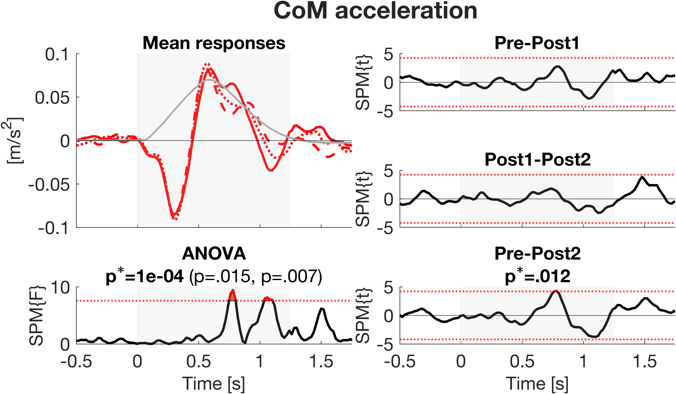 E** | **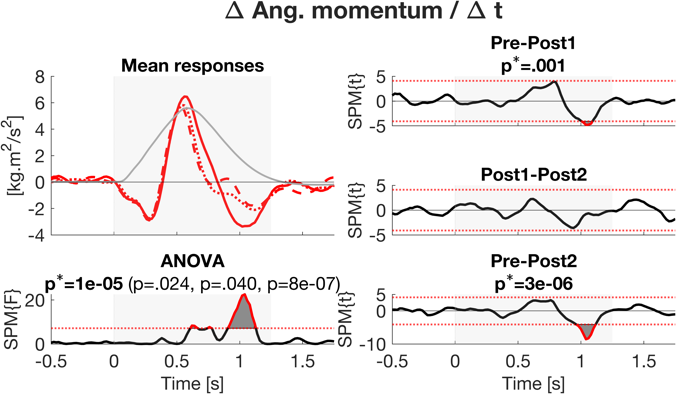 F** |
| **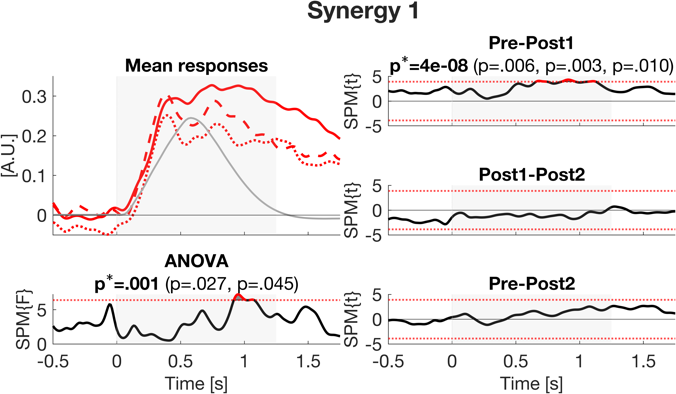 G** | **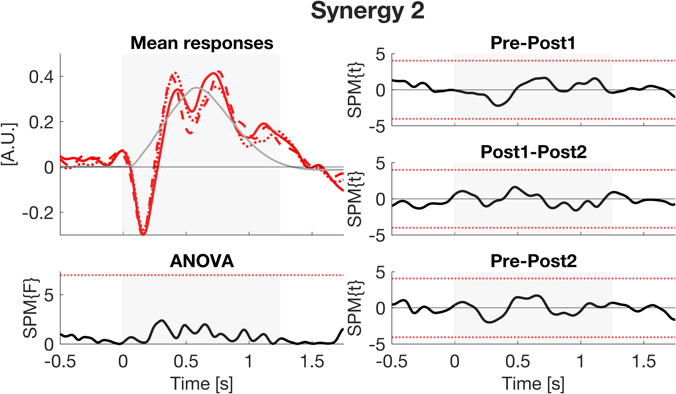 H** |
| **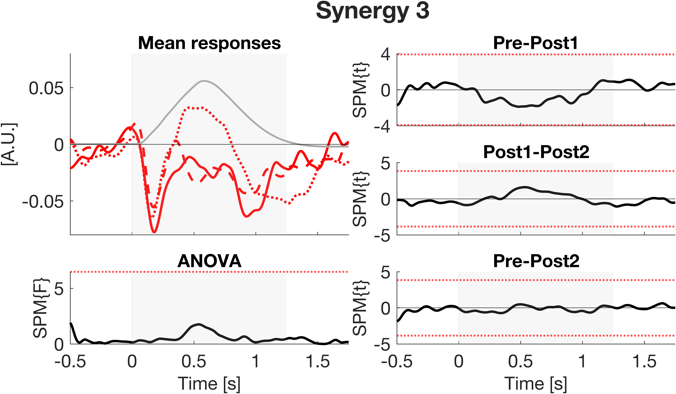 I** | **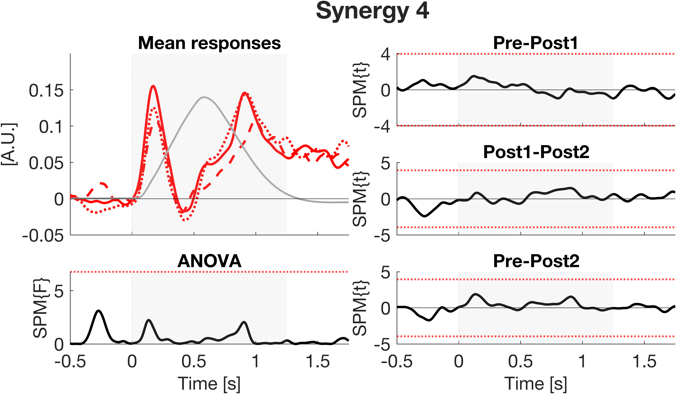 J** |
| 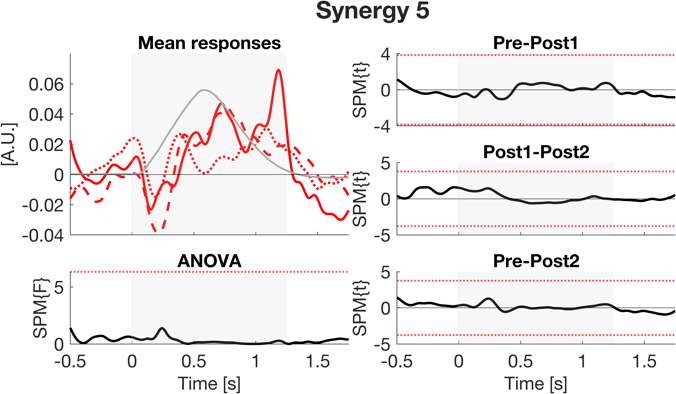 **K** | Figure S3: SPM1d statistics of the recovery responses after lateral perturbation for all parameters. Each panel displays the mean response per session (left top), ANOVA F-statistic (left bottom), and post-hoc t-statistic (right) over time for a single parameter. The degrees of freedom for all ANOVAs and dependent sample t-tests were [2,38] and [1,19], respectively. Effects were significant if the test statistic exceeded the critical threshold (red dotted lines). For the t-tests Bonferroni correction was applied. **p^*^** represent the test’s omnibus p-value. If more than one cluster was present, their individual p-values are stated after the omnibus value in chronological order. |

## **Complete overview statistics – Medial perturbation**

| **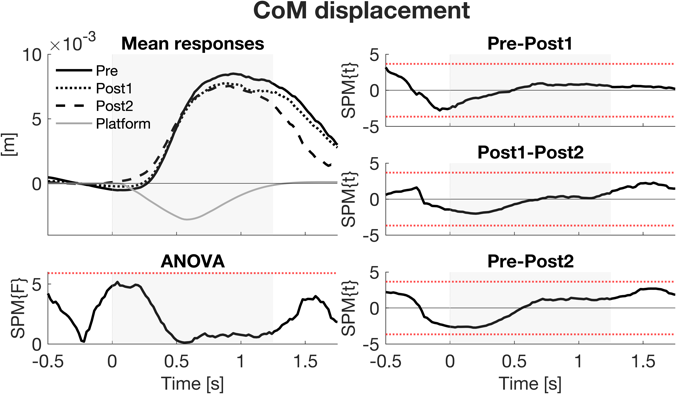A** | **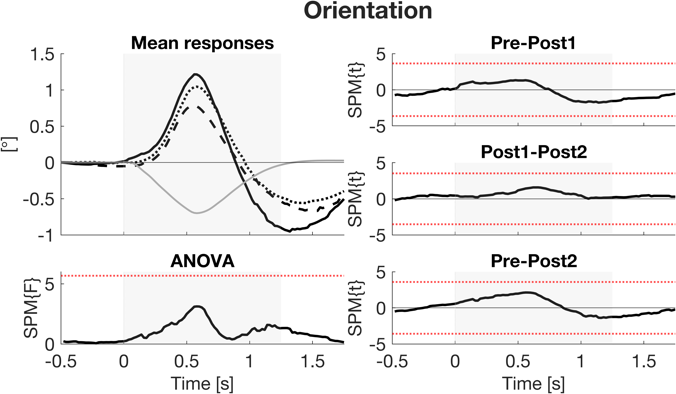B** |
| --- | --- |
| **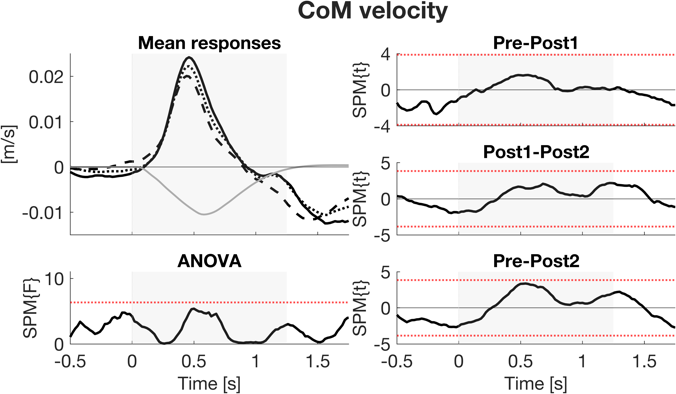C** | **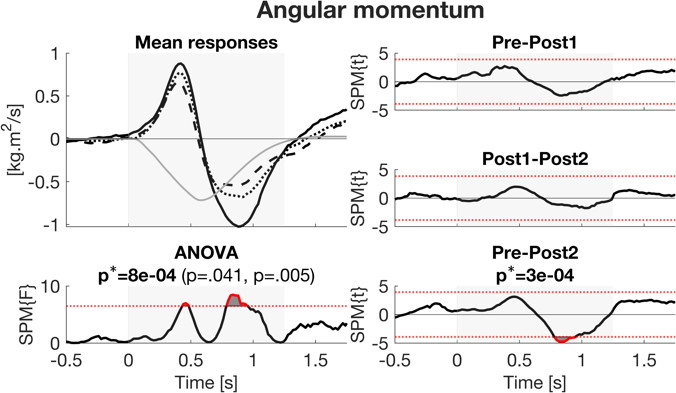D** |
| **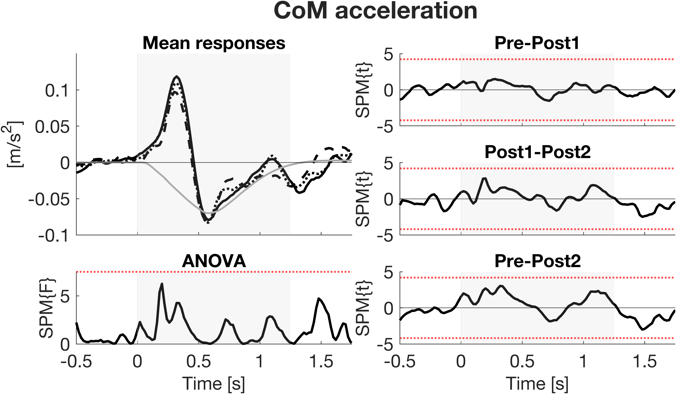E** | **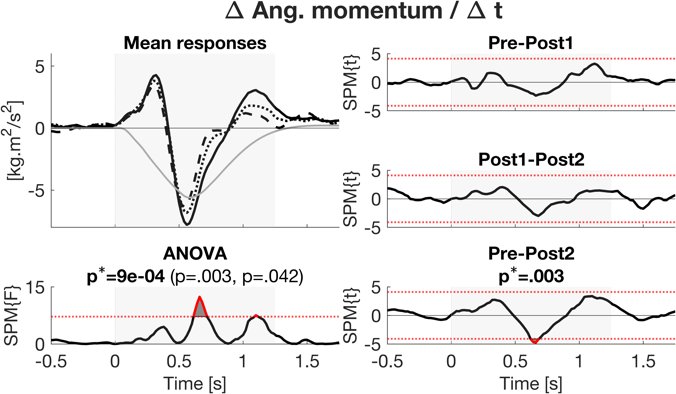F** |
| **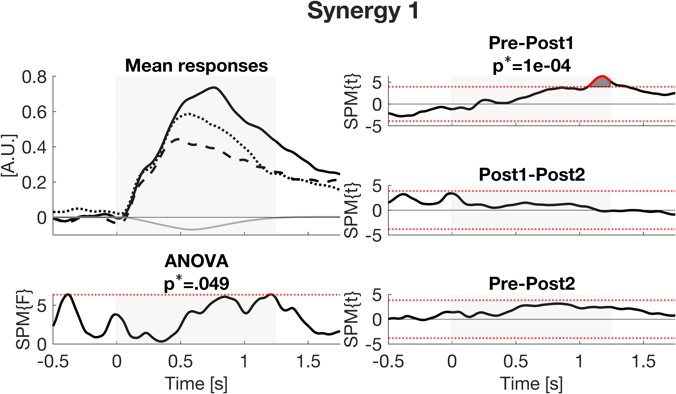G** | **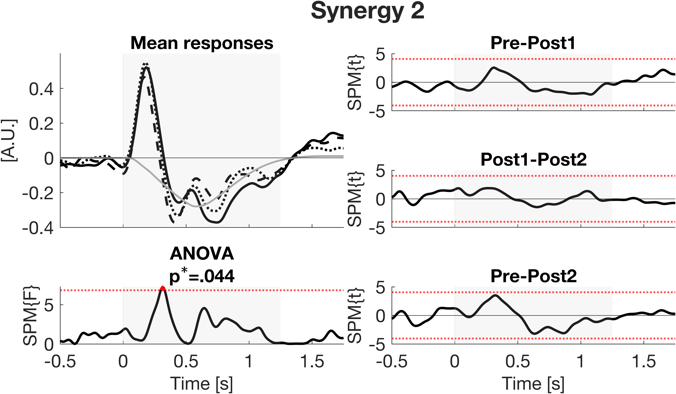H** |
| **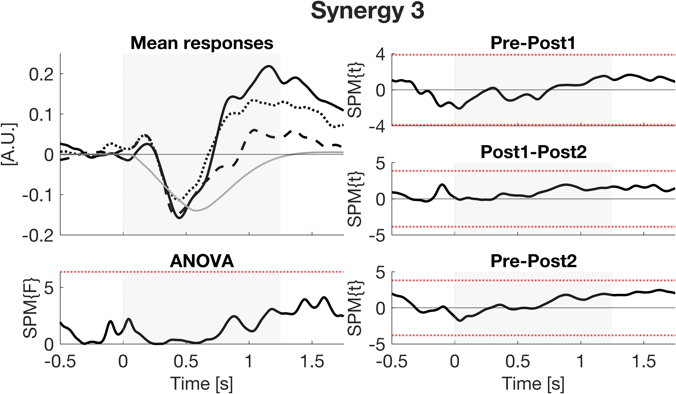I** | **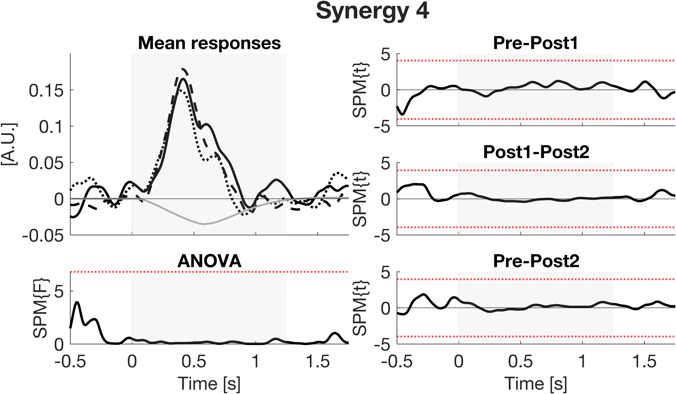J** |
| 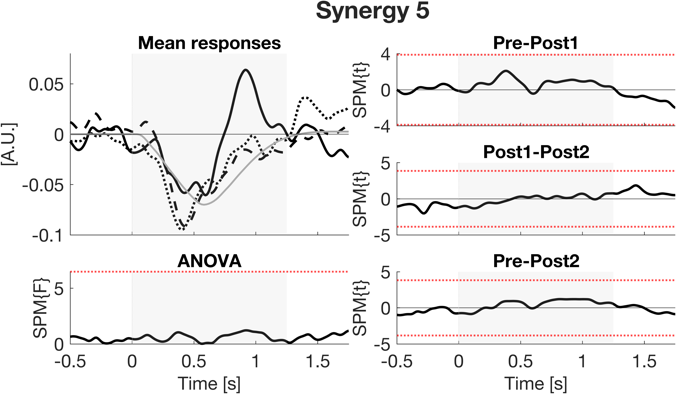**K** | Figure S4: Statistics of the recovery responses after medial perturbation for all parameters. Each panel displays the mean response per session (left top), ANOVA F-statistic (left bottom), and post-hoc t-statistic (right) over time for a single parameter. The degrees of freedom for all ANOVAs and dependent sample t-tests were [2,38] and [1,19], respectively. Effects were significant if the test statistic exceeded the critical threshold (red dotted lines). For the t-tests Bonferroni correction was applied. **p^*^** represent the test’s omnibus p-value. If more than one cluster was present, their individual p-values are stated after the omnibus value in chronological order. |

## **Variability kinematics – Lateral perturbation**

| 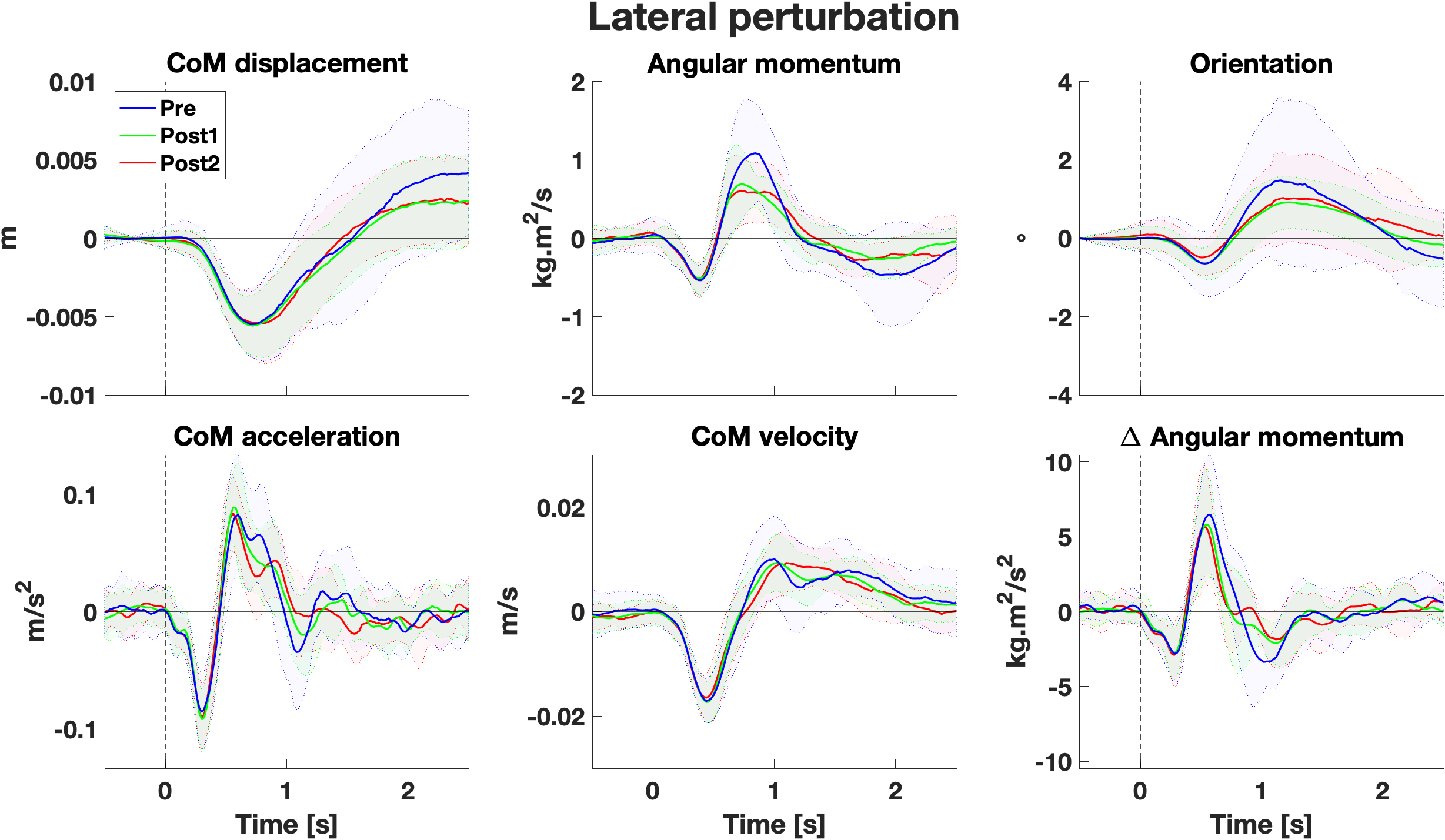 |
| --- |
| Figure S5: Kinematic responses to lateral perturbations. Group mean (solid lines) and standard deviation over subjects (coloured patches) per session. |

## **Variability kinematics – Lateral perturbation**

| 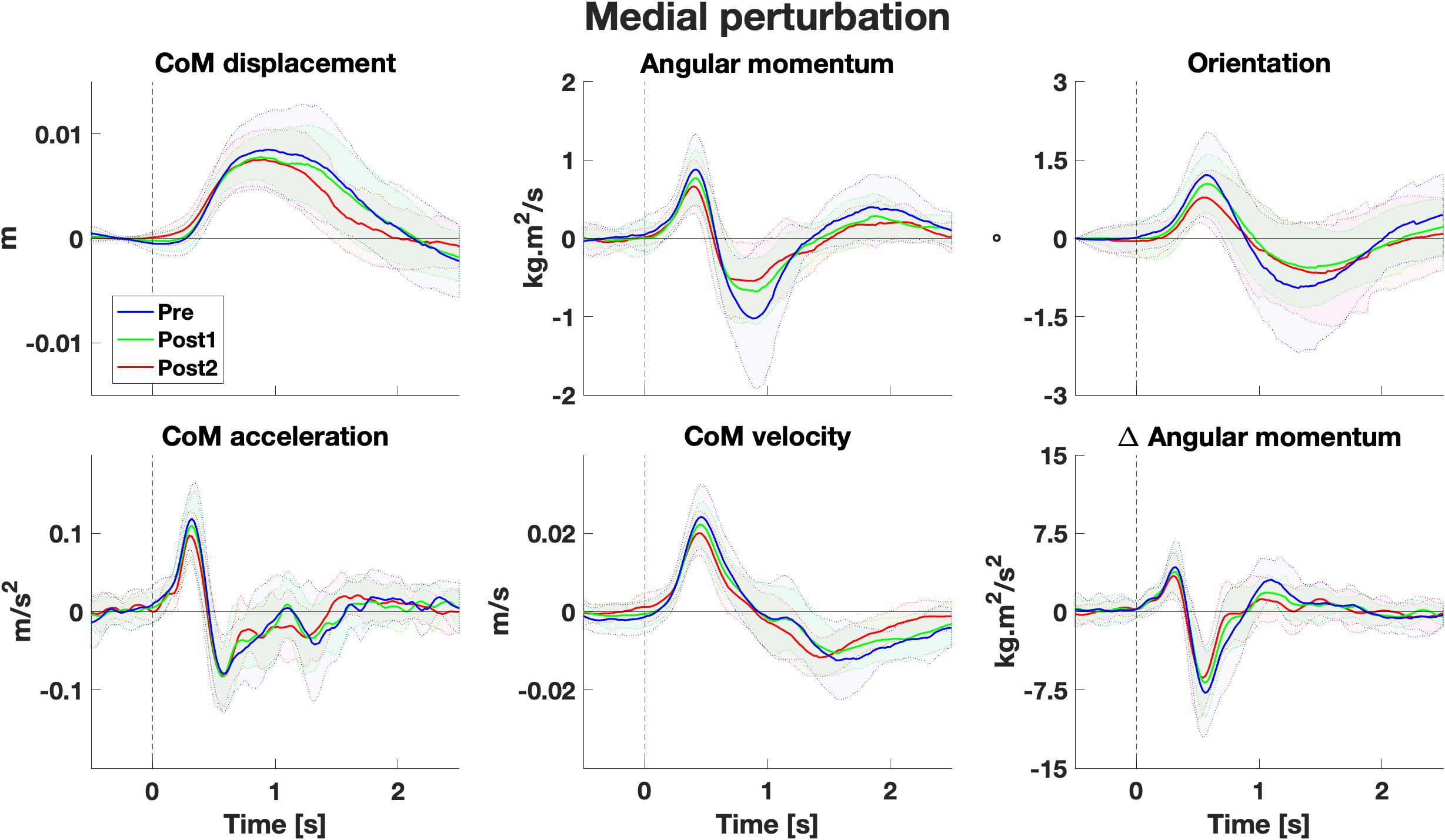 |
| --- |
| Figure S6: Kinematic responses to medial perturbations. Group mean (solid lines) and standard deviation over subjects (coloured patches) per session. |

## **Variability EMG – Lateral perturbation**

| 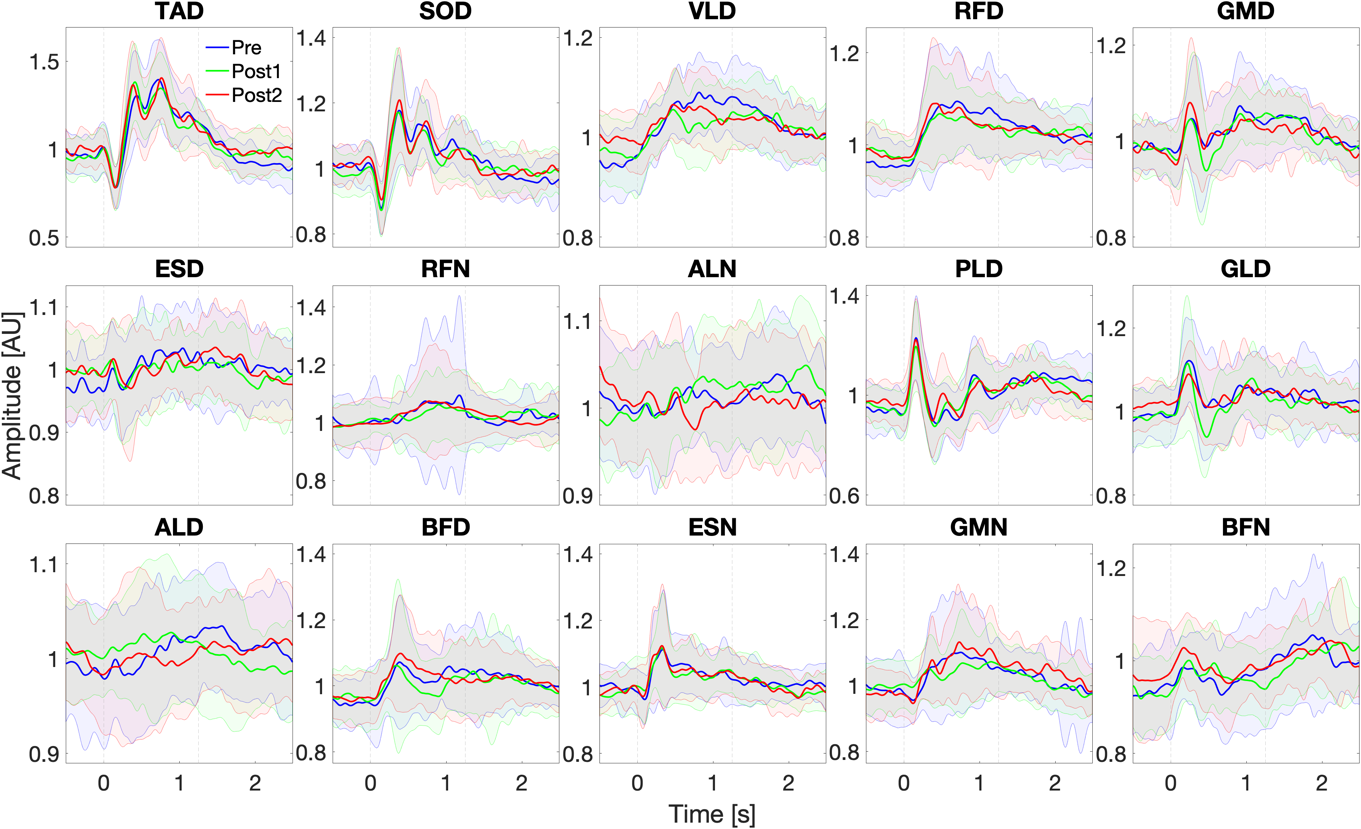 |
| --- |
| Figure S7: EMG responses to lateral perturbations. Group mean (solid lines) and standard deviation over subjects (coloured patches) per session. The letters D & N in the titles indicate muscles on the dominant and non-dominant side, respectively. TA: tibialis anterior, SO: soleus, VL: vastus lateralis, RF: rectus femoris, GM: gluteus medius, ES: erector spinae, AL: adductor longus, PL: peroneus longus, GL: gastrocnemius lateralis, and BF: biceps femoris. |

## **Variability EMG – Lateral perturbation**

| 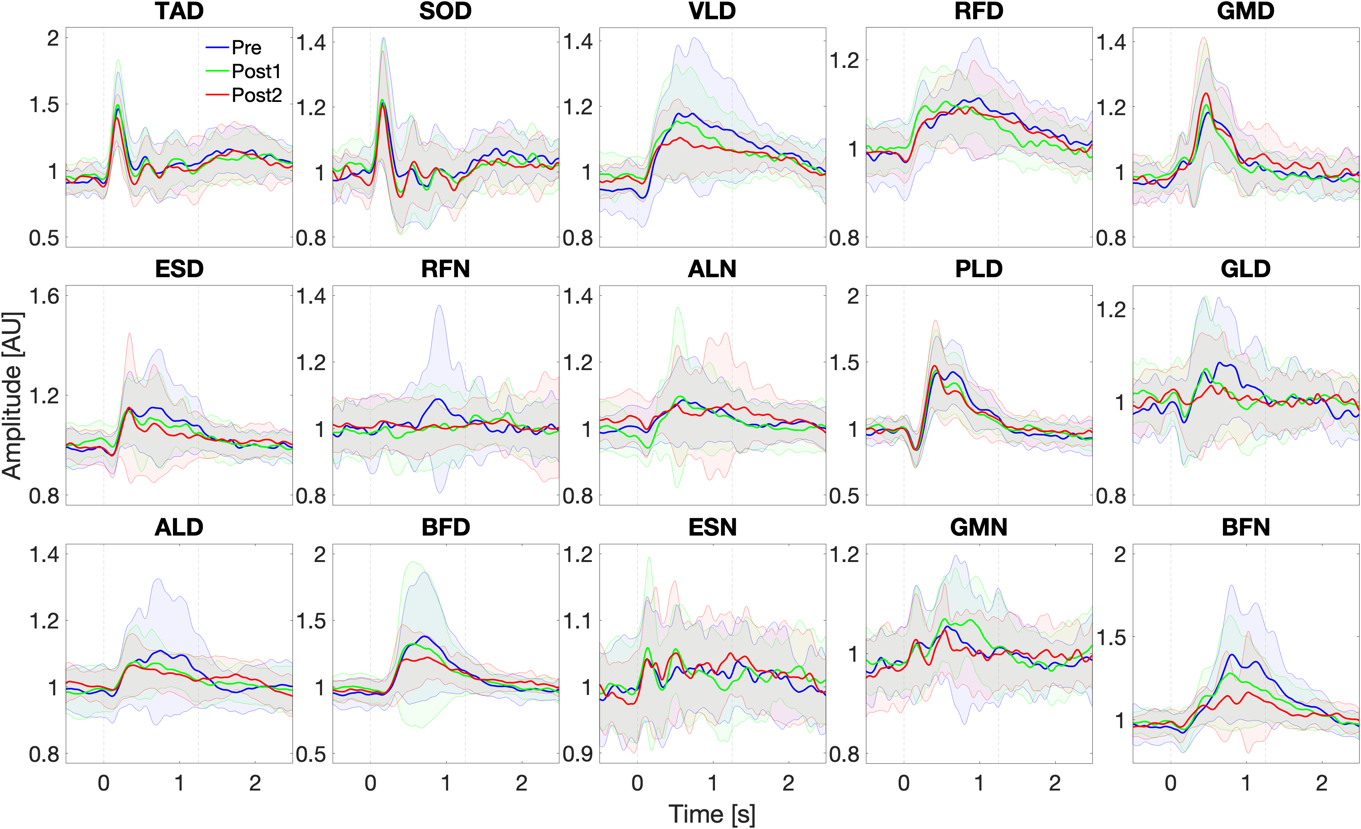 |
| --- |
| Figure S8: EMG responses to medial perturbations. Group mean (solid lines) and standard deviation over subjects (coloured patches) per session. The letters D & N in the titles indicate muscles on the dominant and non-dominant side, respectively. TA: tibialis anterior, SO: soleus, VL: vastus lateralis, RF: rectus femoris, GM: gluteus medius, ES: erector spinae, AL: adductor longus, PL: peroneus longus, GL: gastrocnemius lateralis, and BF: biceps femoris. |
